# The Mentor-Mothers program in the Nigeria Department of Defense: policies, processes, and implementation

**DOI:** 10.1186/s12913-022-08382-8

**Published:** 2022-08-03

**Authors:** Josephine Moshe Ibu, Euphemia Mbali Mhlongo

**Affiliations:** Discipline of Nursing, School of Nursing and Public Health, University of KwaZulu-Natal, Private Bag X5400, DurbanDurban, 4000 South Africa

**Keywords:** Mentor Mother Program, Processes, Implementation

## Abstract

**Background:**

Nigeria has the second largest HIV epidemic in the world and is one of the countries with the highest rates of new pediatric infections in sub-Saharan Africa. The country faces several challenges in the provision of healthcare services and coverage of Prevention of Mother to child transmission of HIV. In the Nigeria’s Department of Defense, prevention of vertically transmitted HIV infections has been given a boost by utilizing Mentor Mothers to facilitate antiretroviral compliance and retention in care. The aim of this study was to explore those processes and policies that guide the implementation of the Mentor Mothers program for PMTCT of HIV in the Department of Defense in Nigeria as no studies have examined this so far.

**Methods:**

The descriptive, qualitative research approach was utilized. We conducted 7 key informants interviews with 7 purposively selected participants made up of 2 program Directors, 1 Doctor, 1 PMTCT focal Nurse, 1 PMTCT site coordinator, 1 Mentor Mother, and 1 patient from one each of the health facilities of the Army, Navy, Airforce and the Defence Headquarters Medical Centre. Open coding for major themes and sub-themes was done. Data were analyzed using thematic analysis.

**Results:**

Findings revealed that the program in the Department of Defense had been modelled after the WHO and implementing partners’ guidelines. Foundational Factors; Leadership; Skill acquisition; and Service Characteristics emerged as processes guiding the implementation of the Mentor-Mothers program in the DoD. These findings supported the Mentor Mother Model, which empowers mothers living with HIV – through education and employment – to promote access to essential PMTCT services and medical care to HIV positive pregnant women.

**Conclusion:**

We concluded that no definitive policy establishes the Mentor Mothers program in the DoD. Working with Doctors, Nurses, local & collaborating partners, and communities in which these hospitals are located, the Mentor Mothers play a pivotal role in the formation, facilitation, and implementation of the MM model to effectively decrease HIV infections in children and reduce child and maternal mortality in women and families they interact with.

## Background

As documented by AVERT.org [1], Nigeria has the second largest HIV epidemic in the world and is one of the countries with the highest rates of new HIV infections in sub-Saharan Africa. This is reflective of the numerous challenges in the provision of healthcare services and coverage of PMTCT of HIV in the country. Since independence in 1960, the Nigerian health system has been confronted with such challenges as maternal mortality, infant mortality, poor sanitation and hygiene, poor disease surveillance, infectious diseases such as tuberculosis and malaria, poor vector control, high incidences of non-communicable diseases and road traffic injuries [2]. Despite recording an improvement in the HIV prevalence of 1.4% among adults aged 15–49 years as compared to the previous estimate of 2.8%, in 2019, some studies show that only 32% of pregnant women living with HIV have access to antiretroviral drugs for PMTCT, with about 12% of HIV-exposed infants receiving HIV testing for early diagnosis by age 2 months in Nigeria [3]. Maximum adherence to ART is of extreme importance in achieving optimal viral load suppression to reduce the risk of mother-to-child transmission (MTCT) [3]. Even so, the inability to effectively address the country’s numerous public health challenges has contributed to the persistent and high level of paediatric new infections across the country.

As indicated by the UNAIDS 2018 data [4], about 36, 000 New HIV infections occurred in children aged between 0-14 years and 45, 000 in young women aged between 15–24 years in Nigeria in 2018. Much as pregnant women and children under five years have been prioritized as beneficiaries of free health policy in some states in Nigeria, the maternal mortality ratio for Nigeria is still shown to be as high as 814 per 100,000 live births according to the 2019 World Health Statistics. [5]. This has been attributed partly to lack of access to affordable healthcare services and workforce shortages in healthcare. To achieve optimal viral load suppression and reduce the risk of MTCT of HIV, the World Health Organization in 2006 recommended “Task Shifting” as a means of initiating and managing more patients to meet the demand for antiretroviral therapy. This meant that the “task” of promoting maximum adherence to ARVs was extended to non-regular medical personnel (to boost manpower shortage in HIV care) such as mentor mothers (MMs). To also accelerate access effectively to PMTCT, such polices as the ‘National HIV/AIDS Policy’, ‘National HIV Strategic plan’ (2010–2015), the ‘National Scale-up Plan’ towards elimination of mother-to-child transmission (eMTCT), the ‘Midwives Service Scheme’, all aimed at ending MTCT were introduced by the Federal Government of Nigeria. [6]. This response was guided by the National HIV Strategic Framework 2014 –2021 [7] the ‘WHO Global Plan’ [8] which aim at terminating HIV/ AIDS by achieving zero new infections, zero AIDS related deaths and stigma. The National HIV/AIDS Strategic Framework consists of 5 thematic areas namely-

- Prevention of HIV among key populations
- HIV testing services
- Elimination of Mother-to-child Transmission of HIV (eMTCT)
- HIV treatment
- Care and support and adherence to ARVs.

These 5 components have been carefully put together as a means of tackling HIV and its related challenges in the country such as stigmatisation and access to ARVs. For instance federal and state laws restrict the disclosure of a person’s HIV status, except in extremely limited cases. This is due to the perceived stigma and discrimination against people living with HIV. In the DoD the patients right to confidentiality is guided by the ‘DoD policy HA 96–034: Request for Medical Records or Medical Information concerning HIV positive Members’. This memo discusses the confidentiality of medical information for HIV positive service members and guides care providers on matters of anonymity and confidentiality. As part of its strategic interventions, the framework also fosters an enabling environment for HIV positive pregnant and breastfeeding mothers and HIV-exposed infants to access antiretroviral drugs [7]. The intervention also includes instituting and strengthening the quality management systems for all eMTCT facilities in Nigeria. To acknowledge the efforts instituted thus far, the Department of Defense (DoD) in Nigeria has keyed into this initiative and provides access to quality healthcare for all eligible military personnel and their families, including HIV prevention services. The Nigerian military exists for the defense of its nation’s sovereignty therefore it is structured to be combat ready both at peace and at war times. Consequently, its personnel are required to be physically, mentally and psychologically fit to carry out these functions. The basic task of provision of medical cover for military operations and exercises and peace support operations at home and abroad is carried out by the individual medical services of the Army, Navy and Air Force as there is presently no unified armed forces medical service. Since MTCT is the main means of peadiatric HIV transmission, recent global efforts are geared towards the virtual elimination of pediatric HIV through PMTCT programs [9, 10]. These programs have proven beneficial as corroborated by UNAIDS report which indicate that PMTCT programs can substantially reduce the risk of vertical HIV transmission from greater than 40% to less than 5% by their ability to help prevent more than about 1.4 million HIV infections among children between 2010 and 2018 [11]. The above was given priority in the DoD in 2014 when it engaged the services of MMs who together with Doctors, Nurses, and PMTCT site coordinators work to provide PMTCT services to newly diagnosed HIV positive pregnant women.

MMs are women living with HIV who have had personal experiences in PMTCT programs (have had HIV-free babies), trained briefly on the basic rudiments of HIV and are willing to use their own personal experience to support other women and their families in HIV care. They act as mentors to diagnosed HIV positive pregnant women within the PMTCT programme to ensure compliance and retention in care [12]. They have been utilized to propagate the concept of the PMTCT programs in the sub-Saharan Africa and beyond. According to Olakunde et.al.[13], though MMs are not engaged in routine Antenatal (ANC)/PMTCT care in Nigeria, the concept of the PMTCT program however began in December 2000 in Nigeria, with the inauguration of the PMTCT National Task Team (NTT) in line with the World Health Organization (WHO) guidelines, while actual PMTCT services commenced as a pilot project in July 2002 [13]. The integrated national guidelines for HIV prevention, treatment and care provides general guidance for the implementation of key interventions for the prevention, treatment and care of people living with HIV (PLHIV) as well as PMTCT [14], therefore interventions engaging MMs for peer support of HIV positive women for linkage and retention in PMTCT has been adopted and implemented in Nigeria since 2007. This came as one of the health sector responses to the HIV/AIDS epidemic in the country [15].

Much as literature demonstrates the beneficial impact of the MM program on PMTCT globally, no structured evaluation of PMTCT or the effectiveness of its services since inception has been undertaken in Nigeria despite the high burden of MTCT of HIV [16], and no published studies have explored the perspective of the MM program implementation in the Nigeria’s DoD. As such, Oleribe et.al [16] note that there exists inadequate data on the MM programs in Nigeria. This is also echoed by Cataldo et al. [17] who further state that the involvement of “mentor mothers” in HIV service delivery has not only created an additional source of human resource for health, especially in low-resource, high disease-burdened settings but is also very effective in the context of rapid antiretroviral therapy (ART), yet details of its evaluation are limited. Asides helping the mothers to remain adherent on their medication, MMs provide additional counselling, education and support to enhance coping skills of pregnant women newly or previously diagnosed with HIV infection. This strategy ensures that women are retained in PMTCT care and treatment as stated by Igumbor et al. [18]. Available records from Mother2mother.Org. [19] confirm that the MM model has been standardized for PMTCT care and implemented in 10 other African countries (within the sub-Saharan Africa) apart from South Africa where it was first introduced. These include Ethiopia, Kenya, Lesotho, Malawi, Rwanda, Swaziland, Tanzania, Uganda, Zambia) and other countries in the globe like the United States and United Kingdom [19]. According to Nokwanele Mbewu, senior programme manager (The Philani Mentor Mothers) programme, evidence has demonstrated that the intervention has significantly improved maternal and pediatric HIV outcomes across cultures. Shroufi et.al [20] also affirm this beneficial impact of peer support on knowledge and attitudes to HIV just as the Nigeria DoD in line with the national HIV strategic framework utilizes MMs in some of its health facilities across the 36 states in Nigeria to meet PMTCT targets and curb peadiatric HIV infections.

## Objective

The objective of this study was to explore the policies and processes that guide the implementation of the MM program in the Nigeria DoD health facilities. This paper is one in a series of five (part of a PhD study) that sought to analyze the implementation of the MM program in the DoD and provides the basis for developing a framework to facilitate utilisation of MMs in other hospitals in the DoD currently not engaging them.

## Methods

### Study design

The descriptive exploratory qualitative design as described in Howitt & Cramer [21] was utilized in the study. Being a novel project in the DoD, this approach was deemed appropriate to explore some national health policies in Nigeria and their influence on the implementation of the MM program in the DoD [22–26]. This rationale presented an avenue to carry out an in-depth description of the DoD MM program implementation and processes, providing analysis and interpretation of findings that were illustrative of the responses of the participants. Seven semi-structured interviews were conducted with seven purposively selected participants made up of two program directors, a PMTCT Doctor, a Nurse, a PMTCT site coordinator, a (n) MM and a patient. Data was collected from the Guards Brigade Medical centre, DHQ Medical Centre, 063 NAF base hospital, and the MODHIP headquarters. This was to ensure inclusiveness as the participants each have engaged at different levels of the DoD MM program. Collectively, their views provided a better understanding of the processes and policies underpinning the DoD MM program. The thematic analysis (TA) technique formed the basis for reporting the findings**.** The manuscript has been developed in accordance with the COREQ Standards for Reporting Qualitative Research.

### Setting

The research was conducted in selected health facilities of the Nigerian Army, Navy, Airforce, the Defence Headquarters Medical Centre (DHQ), and the HIP headquarters in Abuja. Abuja, the Federal Capital Territory (FCT) administratively assumes the status of a state with its own minister who is equivalent to a governor in any of the 36 states. Abuja is in the north central part of Nigeria (middle belt region) and is home to the headquarters of the Nigeria’s DoD with well-established health facilities providing HIV/PMTCT care. The MODHIP coordinates all military HIV programs in Nigeria and is responsible for policy decisions/research in HIV medicine in the Nigerian military. The DHQ Medical Centre acts as the administrative Headquarters and oversees all PMTCT programs in the Nigeria DoD health facilities.

### Participants

Appointments were made with the directors and heads units for the purpose of obtaining permission to access participants and conduct interviews. JMI paid several visits to the proposed participants to get familiaised with them, explain the nature of the research and to gain their cooperation to participate in the study. The maximum variation sampling strategy was adopted for this study. Twelve participants were proposed for the study but due to the contextual nature of the Nigerian military, access to the facilities by non-staff was restricted therefore JMI had controlled access to most participants particularly those at policy level. That accounted for the (seven) number of participants who were eventually chosen from one each of the military health facilities of the Army, Navy, Airforce hospitals, the MODHIP and the DHQ Medical Centre in Abuja.

The participants were purposively selected based on availability, choice to participate in the study and capacity to enhance understanding and exploration of the research question. A well-informed and experienced MM was recruited to facilitate access to the participants. They were approached face to face initially and informed that the research was for the purpose of obtaining a doctoral degree by JMI. Thereafter, leaflets describing details of the research were presented to them and opportunities given to them to discuss the research topic. Subsequent visits were then made to the participants to obtain their consent to participate in the study. Their training, wealth of experience in HIV/ PMTCT care and roles in the MM program were considered for participation in the study. All participants were recruited over a one month period following the initial engagement. Eligibility for inclusion in the study was first the willingness to participate, a minimum engagement period of six months in the MM program either as a patient, care giver or administrator (program director). None of the seven participants selected withdrew from the study.

### Participants characteristics

Of the seven participants, three were male while four were female. They were significant stakeholders in the DoD MM project being that they were all engaged at different levels of the program. The program directors specifically are responsible for policy decisions regarding the MM program and coordinate MM program activities and all HIV/PMTCT services in all the armed forces hospitals across the 36 states in Nigeria. In conjunction with the United States DoD under the Walter Reed Programme, the directors formulate policies, recommend, recruit and train MMs, Doctors, Nurses and other key team members in PMTCT care, as well as supervise the various PMTCT sites in all the armed forces hospitals in the DoD. The Nurses act as focal persons (principal contacts for all PMTCT clients in each of the health facilities). The Doctors provide consultation for all PMTCT patients on a one-on-one basis and treatment options are explored based on each client’s needs. The site coordinators man the various PMTCT centres and also recommend MMs who have successfully benefited from PMTCT in the various DoD hospitals and living healthy for employment. These MMs are considered as good examples to newly diagnosed HIV positive women to aid retention in care. They also complement the healthcare workforce and serve as strong linkages between health care providers and peer support networks. The rationalization of these participants’ personal experiences and the DoD operational activities were employed as a means of harnessing data to understand the processes governing the implementation of the MMs program within the DoD health facilities.

### Instrument

The semi-structured interview guide for this study was developed by the researcher, guided by “The WHO Health Systems Responsiveness Key Informant Questionnaire (2000),” the “Key Informant Survey (2001)” and “The Health system performance assessment (HSPA, 2009) tool” after conducting literature review [27–29]. It consisted of 4 key questions and appropriate probes to explore the research objective and was pilot tested to refine the tool**.**

### Ethical approval

Approval to conduct this study was obtained from the University of KwaZulu-Natal (UKZN) ethical review committee (protocol number 00000186/2009) and the Ministry of Defense Health Research Ethics Committee (MODHREC) (reference number MODREC/APP.312/122/0/7/20/1/9). The Nigerian Ministry of Defence (MOD) operates a joint partnership with the United States (U.S) DoD/Walter Reed Program to provide HIV research and prevention services in the Nigerian military. We observed standard protocols as stipulated in the Helsinki declaration to ensure confidentiality and anonymity of participants throughout the data collection and analysis processes. As much as possible, we made sure our views did not influence our interpretation of the participants, as we did not superimpose our personal understanding of the issues discussed with them.

### Data collection and management

After obtaining gatekeeper permission from the MODHIP, a research assistant was engaged to facilitate identification of`participants and schedule interviews. Participants were informed that taking part in the study was voluntary and that they may choose to withdraw at any point in the research process without any consequences attached. Researcher reflexivity was taken into account in this study. JMI is a nurse with many years of clinical practice to her credit who had interacted severally with HIV clients. However, this interaction and knowledge did not interfere with the process of data collection or influence the report of findings that emerged from the study in anyway. JMI detached herself from the study to objectively identify areas of influence in the study to avoid potential influence on the study process [30]. As part of the obligations to the participants, JMI conformed to those ethical principles spelt out in the Helsinki declaration 2013. She monitored assumptions, preconceived ideas and relationship with participants throughout the research process to minimize any influence on the study results and bias. Thus she constantly reflected on her interaction with participants and ensured that the study finding were based on data provided by the participants.

All interviews were conducted by JMI (the researcher) who is an experienced Registered Nurse currently undertaking a clinical doctoral research but not directly involved with care provision in the MM program. Before each interview, written and verbal informed consents were obtained from the participants and at their choices, the interviews were conducted in their offices during lunch breaks and free from distractions. The one patient (participant) agreed to have her interview in the hospital’s conference room on a day chosen by her. The interviews which lasted between 30–45 min each took the form of an open conversation and were audio recorded with the participants’ permission. Despite the heterogenous predetermined sample size, data saturation was observed among the different cohorts of participants as they all had the same (good) knowledge about the DoD MM processes. All interviews were conducted in a detailed and consistent manner over a period of six weeks. There were no repeat interviews for this study. The research assistant was also present during the interview sessions**.** Measures to ensure rigor were adopted throughout the study. Data were member-checked with participants to verify accuracy at the end of each interview session. Bearing this in mind, field notes were utilised (to keep track of important aspects of my conversations with the participants). Verbal and non-verbal cues were also taken during the interviews to inquire, discuss and explore the research question. These were used during analysis to better interpret the results. Transcribed data were anonymized and reviewed with audio recordings for consistency and accuracy.

### Trustworthiness

In order for the participants to contribute meaningfully during interview sessions, we attempted to establish trust with them by repeated visits to the participants and reemphasizing that participation in the study was voluntary. This was demonstrated in the opening moments of each session by indicating that the study was for academic purpose only and also encouraging them to express their opinions freely. Utilizing the purposive sampling strategy in this study ensured an adequate spread of participants from the facilities selected. To increase trustworthiness, the research process was described in detail, data was coded and compared with relevant literature to enable its replication in other settings when required. To ensure dependability, a detailed documentation of the reflections of events observed during the data collection period was done. An audit trail (to ensure that the findings were based on the participants’ responses) was utilised to describe the research steps up to the presentation of findings. This was to ensure consistency and stability of the data over time and under different conditions By rechecking the emergent themes generated from the data and comparing the data with similar research findings confirmability was assured**.** Finally, the findings were cross-checked with the participants to ensure that the results were a true reflection of their views. This also improved the trustworthiness and strengthened validity.

### Data analysis

A reflexive thematic analysis technique using an inductive approach [30, 31]was utilised for data analysis. Interview transcripts were uploaded into NVivo v.11 (QRS International Ltd). The six-staged approach to TA [32] –(familiarisation with data, assigning preliminary codes to data to describe the content, searching for patterns or themes in the codes across the different interviews, reviewing the themes, defining and naming the themes, and producing the report) utilised in this study focused on identifying, examining and recording patterns in the data and also allowed for extraction of themes from the data set. The transcripts were read several times line by line by the interviewer (JMI), paying attention to and marking important and interesting sections of the transcripts. Initial coding and analysis was done by JMI. A list of initial codes, which formed descriptive labels for naming themes was developed inductively from data- key issues regarding the DoD MM. These functioned as pre-existing categories. Subsequent discussion of the codes with an independent analyst resulted in a consensus on the code names which were later used in generating themes and sub-themes for analysis***.*** The coding list was then updated (deductively) in order to accommodate all emerging themes until a saturation point was reached, implying that no new themes would emerge*.* The codes were then reviewed independently by JMI and analyst and necessary changes made. This was to ensure inter-coder reliability.

**Fig. 1:**
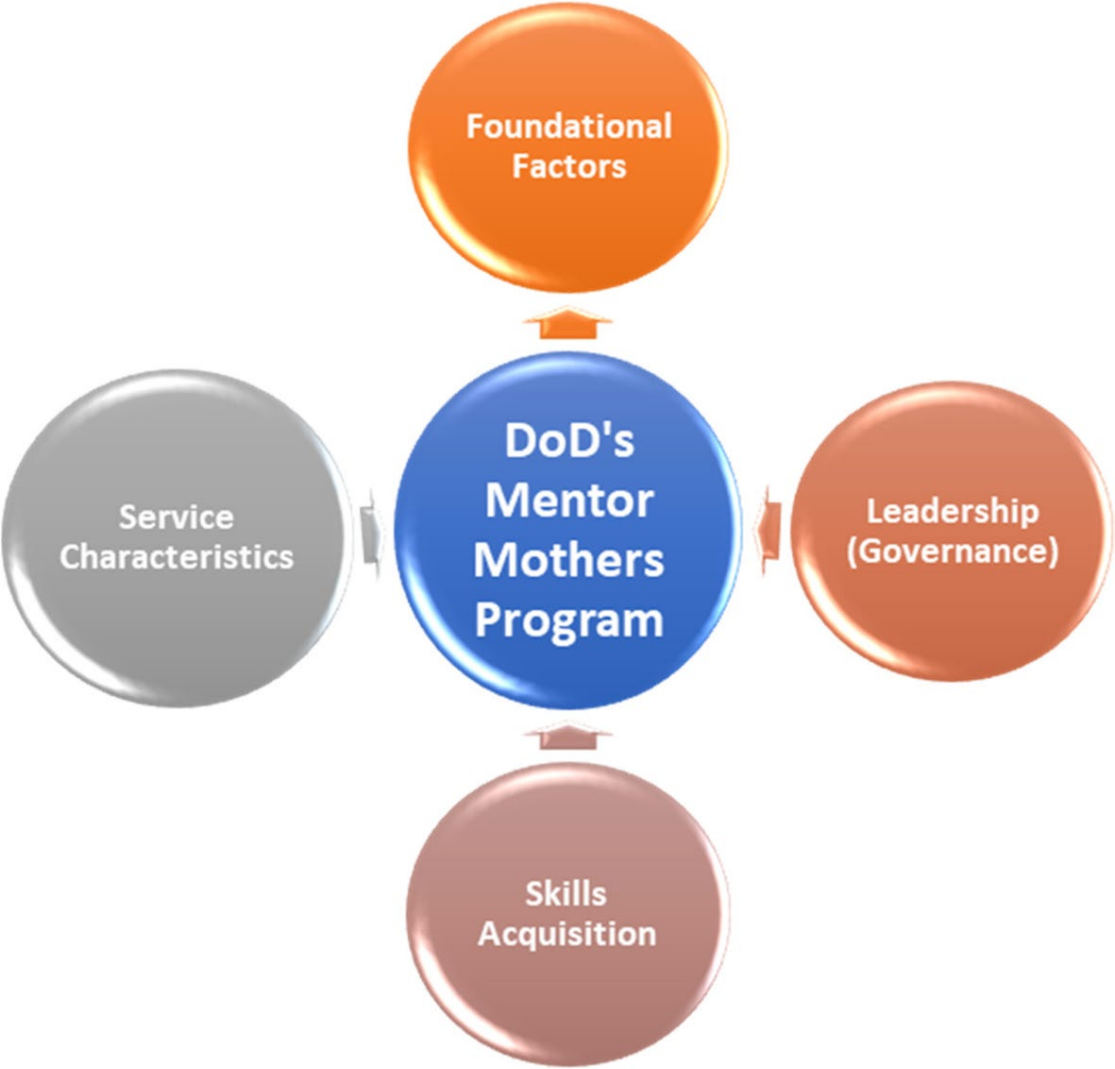
Summary of Categories for the program facilitation and implementation

On completion of coding, similar or related codes were grouped into categories and the categories grouped into themes, which were used in interpreting and reporting the findings. The correlation between each of the themes was identified which explored the views of the participants on the policies and procedures guiding the implementation of the MMs program for PMTCT of HIV within the DoD health facilities. The TA technique [31, 32] demonstrated the relationships between key performance indicators (themes or factors) and their impact on the overall facilitation and implementation of the MMs program within the health facilities. This allowed for individuation of the role of each of these factors in the program implementation and sustainability**.** The participants’ quotes are presented to illustrate each theme and identified by their pseudonyms. Fig. 1 presents a summary of categories that emerged from data.

### Findings

The categories which emerged included foundational factors; leadership; skill acquisition; and service characteristics. These formed the essence for examining the processes and polices that guide the implementation of the MMs program in the Nigeria DoD. The findings also provided insights on the resources and skills needed to successfully manage and sustain the MM program in the various DoD health facilities. The categories, themes, and minor themes (essences) that emerged from data were clearly defined and presented in the Table 1 below:

**Table 1:** Summary of Categories and Themes of the program facilitation and implementation

Categories	Themes	Essence
Foundational factors	Guiding principles/models PEPFAR and State funding Partnership with Nigerian DoD	Program model Funding and key stakeholders
Leadership	Management team support The military Team players/spirit	Management team Management solutions/wisdoms Team spirit or relations
Skill acquisition	Personal experiences with HIV Training Basic educational qualification	Training and Skills Competency
Service characteristics	Promote healthy living Staffing and Wages Social, Financial and Psychological support Service satisfaction	Personnel Services and products Service quality

### Foundational factors

The foundational factors comprised of those policies and processes supposedly guiding the implementation of the MM program in the DoD. This theme emerged through asking some of the stakeholders if there was any document(s) or Act establishing the MMs program. Relevant literature suggested that the National Strategic Framework 2014–2021 and the WHO Global Plan played a major role in the implementation of the MMs program in the Nigeria DoD. The initiative aimed at achieving Zero new infections, zero AIDS related deaths and stigma [8]. While the MMs model has been standardized, established by law and implemented for PMTCT care in Sub-Saharan African countries and around the globe [19], studies have shown that there is no definitive policy or Act establishing the program in Nigeria. Nonetheless, the Nigeria’s National Health Act (No 8 of 2014) Sects. 42 (a-f) [33] empowers the minister of health to create new categories of health care providers to be trained or educated in conjunction with appropriate authority to meet the requirements of the national health system. Though the MMs program is not officially incorporated in the Nigerian National Health Strategic Plan, the Nigeria’s Federal Ministry of Health (MOH) guidelines are an important part of the DoD’ strategy to reduce MTCT of HIV through some mainstreams’ frameworks together with the ‘National Health Act’.[33]. These further support the findings suggesting that through the National Health Strategic Plan, aligned with the national eMTCT Framework and the National AIDS Strategic Plan, the MMs program was developed [33] in the DoD**.** Thus, as a participant in the ‘Global Plan’ on eMTCT, the Nigerian government and by extension the Nigeria DoD also recognize that women living with HIV must be at the centre of the response to the epidemic by critically playing a role in task-shifting to promote health service quality improvement as well as uptake, retention and adherence to care, hence the utilization of MMs for PMTCT services in the DoD health facilities. The foundational factors also revealed three (3) sub-themes which were crucial to the facilitation and implementation of the MMs program in the Nigeria DoD. They included guiding principles/models; PEPFAR and State funding; and partnership with the national health system.

### Guiding principles/models

Guiding principles and models significantly influenced the implementation and shaped the MM program in the DoD [7, 8, 11, 14, 33–37]. All the stakeholders noted that the Nigeria DoD MMs program was modelled after mainstream guidelines/models, such as the WHO guidelines, the ICAP model, National Health Policy, and the Nigerian DoD policy. This was substantiated by the following participants:

*If we are to talk about documents, that was what came up from the World Health Organization. we leveraged on it and together with other implementing partners, we adopted it. (Director B).*

*As I said there is no one document I can pin-point to except from the experiences I came with from ICAP which I proposed to the office and they gave me the go ahead to implement the Mentor Mother Programme (Director Y).*

*The international mentor mother model guides us to be able to fashion out or carve out what we practice in the DoD. It is the same checklist used in accessing the progress.(Doctor).*

*We are guided by the WHO and US DoD guidelines for PMTCT. All our trainings and program activities are fashioned along that line (Nurse).*

From the above submissions, it was evident that there was no definitive guideline underpinning the DoD MM program therefore underscoring the fact that no direct policy or Act guides its implementation in the DoD. However, the DoD policy Document- “DoD Instruction 6485.01: Human Immunodeficiency Virus (HIV) in military service members” updates the policy for identification, surveillance, and management of military personnel infected with HIV and for prevention activities to control the transmission of HIV. Therefore the three Armed forces provide HIV prevention services for both its personnel and their families [37]. Since the main purpose of the MM program is also to promote access to essential PMTCT services and medical care to HIV positive pregnant women, it was necessary to strengthen the existing HIV prevention strategies in place and consolidate on them to achieve safe pre and postnatal outcomes for mothers and babies, and their families alike. One of the directors gave insight into how the program was conceived in the DoD:

*This program started way back in 2015 when we saw the need to improve the services we were providing to the positive mothers living with HIV. We saw a gap first in tracking and the outcome of their babies. We instituted an effective tracking mechanism using people who were lay health workers on ground (Director B).*

By constantly engaging with stakeholders and other implementing partners to explore innovations in PMTCT care, the DoD management strove to expand access to patient centred HIV care for effective service delivery as cited by this participant…

*In one of the review meetings we had with other countries from the east African sub region in Tanzania, experiences were shared….I compared ours to the program elsewhere and that is helping us make improvements (Director Y).*

### PEPFAR and State funding

Funding was one of the foundational factors crucial to the implementation and facilitation of the MM program in the DoD as the findings of this study suggested. In this regard, a partnership exists between the U.S Military HIV Research Program/Walter Reed Army Institute for Research (MHRP-Nigeria) under PEPFAR for primary/facilitative funding sources for HIV activities (through the DoD – PEPFAR funding). The aim of this partnership was to assist the Nigerian government until such a time it eventually takes ownership of the program as the following participants reveal:

*The program is funded by counterpart funding. The DoD brings some part which they administer themselves and the Federal Government of Nigeria makes available some part.(Director B).*

*We have been funded through the Nigerian government in collaboration with the Nigerian Ministry of Defence and the American government through PEPFAR(Director Y).*

In addition, when asked of specific challenges facing the facilitation of the MMs program, the participants indicated that although there had been a joint partnership for funding, these funds have been largely inadequate thereby challenging the progressive implementation of the program. In the current era of gradual withdrawal of funding for HIV by international donor agencies, national governments in affected countries may eventually take over a larger portion of the costs [38]. This is an indication that funding the program in the Nigeria DoD has been very unstable over the years. Thus, expanding the MMs program to other military sites in the country maybe limited by the amount of funds injected into the program over the years. This could threaten the sustainability of the program. This possibility was further expressed by these participants:

*Funding has crashed, you know PEPFAR funding has been dwindling over the years. Otherwise I know they would have said ‘well go ahead and expand to all the other sites’ (Director Y).*

*When there is fund we are able to get personnel, equipment and all the logistics required to keep the program running (Director B).*

### Leadership

Leadership was also noted by the participants as one of the key performance indicators that influenced the facilitation and implementation of the MMs program in the Nigeria DoD. The structure of the program in the DoD made possible by a purposeful leadership adequately equipped the MMs and staff with the needed materials and skills to carry out their tasks. These participants appreciate the responsiveness of the DoD HIV program leadership:

*I think the leadership has been there. Whenever I seek approval to do some things to improve on the program, they allow me to do that. …(Doctor)*

*The leadership, team support and the facilities are running smoothly. The ownership and commitment at the facilities is able to sustain the program so it would not just crash.(site coordinator).*

Thus, this category was associated with sub-themes such as management team support, the military factor, and team player/spirit*.* The essences behind these three (3) sub-themes speak more on the management team, specifically the wisdom and solutions that the management of the program provided, and the team spirit of the sites’ management. These were deemed to be effective factors influencing the implementation of the program. Ensuring unrestricted access to ARVs also served to boost the health of mothers and improved reproductive, maternal, newborn and child health outcomes. Team spirit describes the feeling of loyalty, commitment and pride that exist among members of a team that keeps them committed to the team’s success. This entailed a lot of cooperation among team members. This emerged as one of the key factors influencing the MM program implementation as volunteered by these participants, although that was not without some challenges:

*Due to the team spirit, training and the willingness to sacrifice, our site team commanders give us a lot of encouragement to make sure that the program flourishes (Nurse).*

*In the communities where we operate, the military commanders in the various hospitals, the nurses, every other person is very supportive. That is the spirit behind the success of this program. (Doctor).*

While a portion of the program’s success story was attributed to the management team (including the program staff), a director however reported some lapses:

*My problem is the program staff. some of them wait until consumables are exhausted and they wake up one morning and say there is no test kit. Then they start talking about emergency orders and the rest. (Director B).*

### Skills acquisition

This was considered very significant in the smooth running of the MM program in the DoD. The findings suggested that the program staff acquired skills through three (3) main means: *personal experiences with HIV (as in the case of MMs); training* (through general training on HIV/ PMTCT, review meetings and skills update/workshops and seminars); and *data literacy*. Basic educational qualification (a minimum of secondary school qualification) was a criterion for employment as a (an) MM. The essence of these sub- themes was to measure providers’ preparedness, skills, and competency in their engagement in the MM program. In other words, the findings from this study revealed that to ensure the successful implementation of the program in the DoD necessitated the providers to be adequately trained. For the MMs, the most important primary engagement criteria were personal experience (women who are living with HIV) and have successfully undergone the PMTCT program [39], in addition to having a minimum of a secondary school certificate as it obtains in the DoD. Thereafter, they were trained in several specific aspects related to PMTCT to enable them deliver the right services needed as MMs. Some authors have shed more light on the recruitment criteria and training for MMs in other countries like South Africa [40] which is quite at variance with the kind of training the DoD MMs receive. For instance where the South African MMs are recommended for recruitment by the community and spend a period of six months in the classroom for intense academic work, the DoD MMs are recommended mostly by the site coordinators and undergo basic training. One of the directors explained further:

*It is more of foundational training where we tell them about HIV incidence and how to avoid getting infected; the need for safe breastfeeding because of some people who mix feed, the need for adherence on their drugs and how to follow the mothers up especially those ‘lost-to-follow up (Director B).*

When asked about the criteria for engagement [41, 42] as a MM, the specific training they require for the performance of their duties as well as the contents and nature of their training, some participants responded thus:

*Basically the person must be able to read and write, so a minimum of a secondary school certificate suffices. Although we have some that are Bsc holders. In addition, the person must have a fair knowledge of HIV services, must be a positive woman living with HIV and would have gone through the PMTCT program in the sites (Director Y).*

*First and foremost as a Mentor Mother the issue of stigma should not be in the way. It should be a woman that can come in front of other mothers and say, “I am a positive mother”.(Site manager).*

Findings revealed that the DoD MMs undergo specific training on communication and counselling skills, HIV testing and counselling, PMTCT and paediatric care, adherence to HIV treatment, record keeping, training on confidentiality, infant feeding and child nutrition in line with implementation guidelines to sufficiently equip them with PMTCT skills and provision [17, 42–46], the duration of which is less than a week as volunteered by one of the directors:

*Occasionally they are brought along with the HEADS of Nursing Services in the different health facilities and they are just given a general training on HIV, at least a basic foundational training on HIV for about two days. Some of them come in here for about three days and from time to time they are still invited and updated on the current knowledge (Director B).*

*They receive basic training about prevention of mother to child transmission, HIV testing, disclosure of results to mothers, tracking of lost clients, some elements of EID services, that is early infant diagnosis services. Those are the specific things that are captured in the curriculum we currently operate for them now (Nurse).*

*It is both didactic and practical hands-on training especially when it requires EID services.(Doctor).*

These narratives reveal an absence of a structured curriculum for training of the MMs as it obtains in South Africa. The MM program in the DoD being borne out of the initiative of one of the directors could be one of the reasons for the absence of a definitive policy establishing its implementation and subsequently, lack of a definitive career structure for the MMs.

### Training efficacy(competency)

The essence of training and skills acquisition was to achieve competency in service provision by the healthcare providers, MMs inclusive. This ensured that an efficient human resource workforce was on ground in the health facilities particularly as it concerned HIV care. JMI sought further clarification on the effectiveness of these training programs for the healthcare providers. These participants said:

*The training provided for the staff is good, there are already deployed staff and Mentor Mothers for the program. (Doctor).*

*Sometimes whenever we call them for the review meeting, we also flash on those trainings to remind them especially if there are new things introduced into the program. On the effectiveness I will say it is good because that has improved our indicators for lost- to follow up (Director Y).*

### Service characteristics

Facility and service characteristics predicted access to ART, retention in care and PMTT outcomes in the health facilities and were determined by the DoD health workforce, PMTCT/ART services and quality. Findings revealed four (4) key performance indicators that facilitated the MM program and aimed at promoting healthy living (through tracking patients’ progress, free and inclusive services, EID services, HIV testing; and promoting zero discrimination). These were: *Staffing and wages, service quality*, *financial*, *socioeconomic and psychological support,* and *service satisfaction.* The essences in this category included personnel contribution, services and products accessibility and service quality. The scale-up of ART and PMTCT had been some of the great successes of the MMs program in the DoD since its inception. Therefore as part of its strategic interventions, the MM program fostered an enabling environment for HIV positive pregnant and breastfeeding mothers and HIV-exposed infants to access antiretroviral drugs as substantiated by these participants:

*I access care with DHQ Medical center. I am very close to the mentor mother (Patient).*

*Even on non-clinic days they still access services. If these mothers just bump into us and say, ‘I want to register’, we just attend to them.(Nurse).*

*We run a five-day clinic, throughout Monday to Friday in this facility. There are no special days for ART clinic. They walk in and blend with the outpatients at their convenience (Doctor).*

#### Staffing and wages

Areas worst hit by the HIV epidemic require an ideal number of health workers who are suitably distributed across different occupations and geographical regions to ensure population coverage of health interventions [47, 48]. Health worker shortages in HIV care provision are high in Nigeria which is listed among countries with acute health worker shortages and by extension the DoD. This had been seen to impact on the progress towards reducing the rate of new infections in the country. Asked to describe what guides facility decisions regarding staffing for the MM Program in the DOD, the participants responded thus:

*We felt getting an effective tracker system using the Mentor Mothers who are themselves positive mothers has delivered in our sites… … There are nineteen of them at the moment.(Director Y).*

*The U.S Department of Defense-Walter Reed Army institute of research partners with the Nigerian Ministry of Defense Implementation program to combat HIV/AIDS and public health challenges through training, treatment and research. (Doctor).*

While healthcare workers may earn different levels of salary for instance ‘top ups’, fee-for-service, informal incomes, the DoD MMs are given a stipend irrespective of their academic qualification and period of engagement as cited by one of the MMs:

*Some of us are graduates. The 40,000 Naira has gone a long way to help us but then our desire, dreams are far bigger than that (Mentor Mother).*

The directors justify the current state of the MMs wages:

*We started with about N20 000 and when I presented their issues to the office, they are now paid a maximum of N40 000. We just did it as a program so as to have a unified structure for them.(Director Y).*

*We give them some stipends to encourage them at least for their transport, for making out time to follow up on the mothers who are challenged. Some of them get as much as N40 000, whereas graduates are not even getting up to twenty thousand. So, it is really big, and for some of them who have lost their husbands, this has been a source of income…they are very happy as far as I am concerned.(Director B).*

It would appear that definitely, the MMs exist in the DoD to fill a gap based on an existing need. Therefore when asked about their employment status, this participant described it as ad hoc:

*They are not permanently employed; they are more of *ad hoc*, contract *ad hoc* staff. As far as the program lasts and there is funding, they will continue to exist* (*Director B*).

An interaction with the MM confirmed the narrative above:

*As a mentor mother you are not employed, and because you are not employed you have not tendered any certificate. I cannot tell you whether our employment is contract or casual. (mentor mother).*

Much as complaints such as these had attracted an increment to their stipends, a DoD program director reemphasized the status of their employment:

*Being a mentor mother is a privilege and not a right.. it is not a regular job and they are not coerced (Director B).*

The above narratives underscore the lack of permanency of the MM’s employment which perhaps MMs view as a lack of support*.* However, this participant reveals a role extension for the MMs despite their poor remuneration and job status:

*Basically, we are exposing them to also do more of tracking among people on treatment and data recording around EID services (Director Y).*

Clearly, from these narratives, tracking was a focal aspect upon engagement as an MM. Interestingly, they were also trained on data recording which is a more engaging aspect of the MM system and not only affords research capacity building but also enabled the MM’s to witness how their involvement contributed more comprehensively to the programme. The DoD management also availed patients and other healthcare consumers with social support services to further build capacity towards sustaining the program:

*There are other social services beside HIV services and we’ve always encouraged patients to go to the right place to seek those services. (Nurse)*

*Occasionally we make available little things, there is this OVC (orphans and vulnerable children’s) site where we have a program that takes care of about 250 OVCs and those ones who are out of school. We send them for skills acquisition. (Director B).*

This participant explains further the existence of the MM program and its services:

*The mentor mothers program has come to be because we want to use mentor mothers to encourage other people who have these HIV challenges so that they can adhere to their treatment and other processes that are supposed to keep them and their families healthy (site coordinator).*

Findings also indicated that access to the services within the program was extended to the civilian population as well, as cited by this participant:

*About 85/90% of our clients are civilians. That is just to tell you our services are very accessible (Nurse).*

*All our services are at no costs. PEPFAR supports everything…they are very accessible. (site coordinator).*

#### Service quality

Quality determines the extent to which a product complies with a set standard. The core of the MM program was the ability of the health system to enhance performance standards and draw attention to and strengthen areas of weakness to expand access to anti-retroviral therapy with positive outcomes for both mothers and babies. To achieve this, trained MMs within peer group settings provide individual support for HIV-positive pregnant women and postpartum mothers to help them address unmet needs for understanding HIV, self-care, infant care, psychosocial support, and acceptance, and meeting economic needs over the longer term [48, 49]. On how the recipients of the program would describe the services they received, the participants concluded thus:

*I think they see it that the program has worked. It has impacted them positively and they are happy. They feel the program has been worthwhile and met its goals. (Director B)*

*I think the mothers are happy for it. (Director Y).*

*The mothers see it as a welcome service and these services have actually improved their engagement with the facility. (Doctor).*

These views were summarized by these participants:

F*rom my own assessment I will say to a reasonable extent they will give it a pass mark (Mentor Mother).*

*We have more people being retained in our care now compared to what it used to be (Nurse).*

*“The program has helped some of us live well” (patient).*

## Discussion

The current study critically explores the processes and policies guiding the implementation of the MMs program for PMTCT of HIV in the Nigeria’s DoD and highlights the core components, resources and skills needed to realize organizational goals. Previous studies indicate that although the MMs program is not legally supported by policy in the country, the MM initiative for PMTCT exists in Nigeria since 2007 currently guided by the National HIV/AIDS Strategic Framework [14]. In another study by Sam-Agudu et al. [36], it was revealed that the MM initiative in Nigeria also operates in line with the WHO global plan at different levels of training and structure in some states of the federation such as Kebbi and Sokoto. Primarily the DoD MM program strives towards achieving the goals outlined in the UN/WHO Global plan which ultimately is to get to zero new HIV infections, zero discrimination and zero AIDS-related deaths in pregnant mothers and their babies. In accordance with the Fed Min of Health National HIV/AIDS Strategic Plan, pregnant women were captured at the earliest opportunity and tested during antenatal care, labour and delivery as an important entry-point for PMTCT services [7].

### The DoD’s Mentor Mothers program

The findings from this study revealed that the DoD MM program was also an initiative of one of the directors (director Y) in 2014. According to director Y, he proposed some of the ideas he came with from ICAP to the DoD management and was given the ‘go ahead’ to establish the MM program. Data made available by the Nigerian Military HIV Research Program [37] indicate that MM activities commenced in the 1st quarter of 2014 but took effect in the 2nd quarter of 2015 (in selected hospitals) such as the DHQ Medical Centre, 063 NAF (Nigerian Air Force) Base Hospital – Abuja. The Walter Reed Military HIV Research Program (MHRP)-Nigeria (in partnership with the Nigeria DoD) records show that 3 levels of health facilities exist for PMTCT service delivery in the DoD which comprise 27 comprehensive sites, 11 satellite sites, and 75 outreaches [37]. The comprehensive sites are likeable to tertiary level facilities. the satellite sites represent secondary level facilities while the outreaches are those areas where the MMs meet with their clients and larger community to exchange information and address issues of concern that may arise. This arrangement was as a result of the high burden of MTCT among the military community and its environs. The MM initiative was to compliment the human resource workforce in HIV management to boost target achievement in PMTCT care using MMs [37, 49]. Therefore, working with governments, local and international partners, and communities played a pivotal role in the formation, facilitation, and implementation of the mothers2mothers’ (MM) model [50] in the DoD. This model was to effectively decrease HIV infections in children, reduce child and maternal mortality, improve the health of (women, adolescents, and families), reduce stigmatization and discrimination, and support the livelihood, development of women, families and communities [37, 42]. As related by some of the participants, the DoD presently engages the services of 19 MMs spread across the 3 level facilities in some of its PMTCT sites. Program coordinators, together with the MMs, monitor outcomes like rehabilitation rates over time, uptake and participation in PMTCT; and these outcomes are used to measure the commitment of the MMs and by extension the effectiveness of the program. Findings also revealed that due to a shortage of MMS most of the PMTCT sites are without this category of staff. In such sites, PMTCT services have largely been provided by nurses and other field coordinators. It was also revealed that in some of those sites where MMs function, their roles have been extended due to human resource shortfall and this has been misunderstood by some staff. The resultant effect is role diffusion which previously was envisaged as purely supportive in nature. This does not mean the MMs are not capable, (they have been trained). However, in terms of standard M2M policy they are expected to work in close collaboration and supervision by clinical staff (Nurses as focal persons, Doctors and site coordinators) [17]. Compared to the South African MM program, the DoD MM program differs in some aspects. Being a well-established programme, the South African MMs receive annual refresher training and become references for the doctors and nurses with whom they work. They are paid salaries equivalent to those of community health workers [39]. This is at variance with what currently obtains in the DoD**.** Here the MMs receive training on basic communication and counselling skills, PMTCT, record keeping, confidentiality, infant feeding and child nutrition and must work under guided supervision. They are equally handed stipends which are barely enough to take home.

Gureje et al. and Ubochi et al., [50, 51] draw our attention to the shortage of virtually all cadres of health care workers in Nigeria, leading to poor utilization of most health facilities for essential services in the country, ranging from HIV, ANC to other basic services. This prompted the establishment of the Nigeria’s National Health Act (No 8 of 2014) Sects. 42 (a-f) empowering the minister of health to create and train new categories of health care workers to meet the requirements of the national health system [33]. The DoD management also leveraged on this provision to engage MMs for the propagation of PMTCT care and services in its health facilities. Here the MM program serves as a means of strengthening the quality of the existing management of the health care system for all elimination of MTCT (eMTCT) in its health facilities. Even as the program is fully functional at the various sites, information on its implementation is inadequate. The findings from this study suggest therefore that there are no direct policies or Acts establishing the MM program in the DoD. Notwithstanding, the MMs model has been standardized for effective PMTCT services delivery in the DoD health facilities. The participants relate that this has boosted the workforce shortage as well as bridged the existing gap in HIV care to achieve target goals. Specifically they admitted that the MM program in the DoD had been modelled after the WHO and implementing partners guidelines to suit the needs of the Nigerian military PMTCT program. Similarly, one of the directors stated that he consolidated on his experiences and gains from ICAP which guided him on the initiation and implementation of the MM program in the DoD. Much as this program is not legally backed by a definitive policy or officially incorporated in the Nigerian National Health Strategic Plan, this initiative exists in Nigeria since 2007 and volunteers coordinate public health interventions for linkage and retention of HIV positive women in PMTCT and treatment services. These findings are in line with the views of Olakunde & Adeyinka [13] which indicate that the PMTC initiative in Nigeria has been modelled after the WHO and implementing partners’ guidelines. Again, no structured evaluation of the PMTCT or MM program initiative in Nigeria has been undertaken since it began [36]**.** Similarly, available records indicate that the last comprehensive assessment of the DoD MM program was conducted 2017. The unavailability of progressive evaluation was unanticipated and reflected the lack of a definitive monitoring and evaluation blue-print for the MM program in the DoD. In the same vein, Oleribe & Enenche [16] lament the inadequacy of data on the MM program effectiveness in Nigeria which also reflects the manpower personnel and infrastructural challenges that not only confirm but highlight the numerous gaps and the lack of definitive policies guiding the implementation of the MM program particularly in resource-limited settings.

The current study having examined the MM program in the DoD revealed 4 institutional categories (Foundational factors, leadership, service characteristics, and skill acquisition for service providers) which formed the bedrock for the initiation and running of the program, even as no definitive policy governing its implementation exists (in the DoD). Hence, the findings from this study support the mothers2mothers’ Mentor Mother Model, which empowers mothers living with HIV – through education and employment – to promote access to essential services and medical care to other women [39]. Just like the National Guidelines for the Kenya Mentor Mothers Program (KMMP) were established through the participatory and consultative processes drawn from expert opinions from public health institutions, academic intuitions, NGOs, and development partners [34], the findings from this study are in line with the views of Vrazo et. al [48] and show that leadership support such as government support, experts’ support, institutional support (such as health, academic and private developmental institutions), local and international partners, and communities played a pivotal role in the formation, facilitation, and the implementation of the MMs program in the DoD. Although findings from this study and other studies [36, 40] suggest that the MMs program is not nationally implemented in Nigeria, the development and implementation of the program in the DoD incorporates best practice approaches from PMTCT implementers across the country and internationally for the development of the Mentor Mother Model. These underscore the implementation bottlenecks of the PMTCT/MM programs in Nigeria. Notwithstanding, by identifying the specific needs of the Nigerian military as it concerns HIV/AIDS prevention, the Nigeria DoD has leveraged on the gains of the program globally and in other sub-Saharan African countries to plan and institute HIV prevention/PMTCT programs in its health facilities. The need to ensure all HIV positive pregnant mothers are reached and able to access care and all HIV exposed infants (HEIs) screened early enough to receive care and treatment however remains expedient.

### Study limitations

To begin with, the interviewer (JMI) had controlled access to the military health facilities to access the participants. Perhaps the highly regimented and intelligence guarded nature of the Nigerian military was responsible for this and contributed to the limited sample size. A well-informed/experienced research assistant in one of the health facilities was recruited to facilitate access to the health facilities and participants. Much as information about HIV is available in the public domain, research on engaging MMs for PMTCT in the DoD is limited because there is a non-disclosure policy on HIV matters in the military except where absolutely necessary. This probably accounted for the participants’ exercise of caution with volunteering certain information particularly when it had to do with policy. Although they had been assured that this study was strictly for academic purpose, the use of a tape recorder during the interviews may also have added to their exercise of caution.

It was therefore challenging to get most of the participants to provide detailed information on some critical aspects of the MM program implementation and sustenance. This leaves the potential for the views and perceptions of the major stakeholders at policy/implementation levels to be missing in this study. Again, due to the dearth of previous published works on the MM implementation in the DoD, generalizability could not be made of the findings of this study. Despite these limitations however, various measures to ensure rigor were adopted throughout the study. Field notes were used (to keep track of important aspects of my conversations with the participants). Verbal and non-verbal cues were also taken during the interviews to inquire, discuss and explore the research questions. The exploration of the policies and processes guiding the implementation of the MM program in the DoD highlighted critical implementation gaps. Findings (which were member-checked with participants to verify data accuracy) were based on participants’ views as evidenced in their reported direct quotes. A strength of this research is in the design and the variation in the sample which comprised of different cohorts and allowed for the exploration of a broad spectrum of complexities while also allowing a deeper understanding of the novel MM program in the DoD. Details of this study are also presented so that the study could be replicated. As a high HIV burdened country, Nigeria is yet to cover much ground in its journey towards elimination of MTCT (eMTCT) of HIV. The MM program for PMTCT is one of those strategies by which target achievements for eMTCT could easily be attained. The DoD management could therefor contribute towards achieving the 90–90-90 goals (even in 2022 presently) for pregnant and postpartum women in Nigeria through collaboration, training, and funding for research.

## Conclusion

This study has explored the policies and processes that govern the implementation of the MM program and concluded that no definitive policy exists either in Nigeria or the DoD that establishes the MM program. Some of those processes that have in one way or the other shaped the MM initiative in Nigeria and by extension the DoD support the Mentor Mother (MM) Model which promotes access to essential services and medical care to HIV positive women and their babies. In the DoD, the program has not been without some challenges which bother majorly on funding and sites maintenance. Data revealed that not all the sites in the DoD were covered by mentor mothers. However, the critical implementation gaps identified could mitigate the achievement of the 90–90-90 goals for pregnant and postpartum women in Nigeria even as others have surpassed [24].

### Recommendations

The peer mentorship strategy for PMTCT is a laudable initiative and requires a more formalized, well-defined position that is responsive to the needs of recipients to properly position it in the national health system as suggested by Ubochi et al. [51]. Further research to explore the processes and policies that establish the MM strategy in Nigeria and the DoD should be conducted. To address stigmatisation of people living with HIV/AIDS (PLHIV) in Nigeria, interventions to protect the interests and wellbeing of patients and staff should be developed. The media should be engaged for sensitisation and PLHIV should be involved in service delivery. Most importantly, community mobilisation is necessary.

## Data Availability

The datasets for the current study are not publicly available as this is meant for this research specifically. However, Data can be available upon reasonable request to the authors.

## Abbreviations

ART: Antiretroviral therapy
ARVs: Antiretrovirals
DoD: Department of Defense
eMTCT: Elimination of mother-to-child transmission
ENHAT–CS: Ethiopia Network for HIV/AIDS Treatment, Care and Support
FCT: Federal Capital Territory
HIV: Human immunodeficiency virus
KMMP: Kenya Mentor Mother Program
MHRP: Military HIV Research Program
MM: Mentor Mothers
NMOD: Nigeria Ministry of Defense
OVC: Orphans and vulnerable children
PEPFAR: President’s emergency Plan For Aids Relief
PMTCT: Prevention of Mother-to-child Transmission
SPS: Senior Programs Specialist
WHO: World Health Organization
WRAIR: Walter Reed Army Institute of Research
